# Global trends and future perspectives in autism spectrum disorder and gut microbiota research: a comprehensive bibliometric analysis

**DOI:** 10.3389/fnins.2026.1607951

**Published:** 2026-02-06

**Authors:** Guojun Liu, Linzi Chen, Maosen Guan, Ningkun Xiao

**Affiliations:** 1 Inner Mongolia Key Laboratory of Life Health and Bioinformatics, School of Life Science and Technology, Inner Mongolia University of Science and Technology, Baotou, China; 2 Department of Integrated Traditional Chinese and Western Medicine, The Second Xiangya Hospital, Central South University, Changsha, Hunan, China; 3 Central Plains Wushu Research Institute of Henan University, Kaifeng, China; 4 Department of Immunochemistry, Institution of Chemical Engineering, Ural Federal University, Yekaterinburg, Russia; 5 Laboratory for Brain and Neurocognitive Development, Department of Psychology, Institution of Humanities, Ural Federal University, Yekaterinburg, Russia

**Keywords:** autism spectrum disorder, bibliometric, gut microbiota, gut-brain axis, microbiome

## Abstract

**Background:**

Autism spectrum disorder (ASD) is a heterogeneous neurodevelopmental condition. Increasing studies examine whether gut microbiota alterations and the gut–brain axis are linked to ASD-relevant phenotypes. As the literature expands rapidly, a quantitative mapping is needed to clarify influential work and evolving themes.

**Objective:**

To map global research on ASD and the gut microbiota, identify major contributors and knowledge bases, and characterize thematic evolution and emerging fronts.

**Methods:**

We analyzed 1,391 English-language articles and reviews indexed in the Web of Science Core Collection (1999–2024). CiteSpace, VOSviewer, and R were used to evaluate publication trends, collaboration networks, co-citation structure, keyword clustering, and burst detection.

**Results:**

Publication output increased slowly before 2010 and accelerated after 2018. The United States and China were leading contributors and key collaboration hubs. The co-citation core was anchored by landmark experimental and translational studies, including work on microbiome-to-behavior links and microbiome-targeted interventions. Keyword clustering and timeline views highlighted three prominent thematic directions: fecal microbiota transplantation, Rett syndrome, and maternal immune activation. Recurrent and burst keywords emphasized the gut–brain axis, short-chain fatty acids, gastrointestinal symptoms, and oxidative stress. Recent burst terms, including obesity, major depressive disorder, and glutamate, suggest increasing connections to metabolic and broader psychiatric dimensions.

**Conclusion:**

ASD–microbiome research has shifted from descriptive comparisons toward mechanism-oriented and intervention-relevant questions. Future progress will benefit from standardized protocols, longitudinal designs, and multi-omics integration, together with rigorously designed trials to evaluate microbiome-targeted strategies.

## 1 Introduction

Autism spectrum disorder (ASD) is a heterogeneous neurodevelopmental condition characterized by impairments in social communication, restricted interests, sensory differences, and repetitive behaviors (Xiao et al., 2024a,Lord et al., 2018). These features can substantially affect learning, daily functioning, and overall well-being. Despite progress in behavioral and pharmacological management, the etiology of ASD remains incompletely understood and likely involves interactions among genetic susceptibility, environmental exposures, immune dysfunction, and gut-brain processes (Lord et al., 2018; Lord et al., 2020).

Increasing evidence links the gut microbiota to ASD-relevant physiology and symptoms (Wan et al., 2024; Vuong and Hsiao, 2017). Early studies reported frequent gastrointestinal comorbidities and microbial alterations in individuals with ASD (de Angelis et al., 2015; Williams et al., 2012). The gut-brain axis provides an integrative framework to interpret these observations through neural, immune, endocrine, and metabolic pathways (Mayer et al., 2022; Prince et al., 2025). Recent work has increasingly examined microbial metabolites and immune mediators as candidate mechanisms. Examples include short-chain fatty acids and proinflammatory cytokines, which have been discussed as potential mediators of neuroinflammation and synaptic function (Fung et al., 2017; Morris et al., 2017; Dalile et al., 2019). The literature also suggests that microbial perturbations may relate to neurotransmitter-relevant pathways and inflammatory signaling in ASD (Długosz et al., 2025).

This research area is expanding rapidly and has become highly interdisciplinary. Evidence is distributed across clinical cohorts, animal models, and multi-omic profiling, and analytic pipelines vary widely. As a result, it is increasingly difficult to track influential contributors, map the evolving knowledge base, and identify emerging research fronts using narrative synthesis alone.

However, a structured bibliometric overview of this rapidly growing literature remains limited. Bibliometric analysis can address this need by quantitatively mapping the scientific landscape. It can summarize publication growth, collaboration networks, and knowledge structures, and it can detect hotspots through co-citation and keyword analyses. In this study, we conduct a bibliometric mapping of ASD and gut microbiota research published between 1999 and 2024 based on the Web of Science Core Collection. Using CiteSpace, VOSviewer, and R, we aim to identify major contributors and collaboration patterns, characterize core thematic clusters, and highlight evolving topics that may guide future mechanistic and translational research.

## 2 Methods

### 2.1 Data source and search strategy

We used the Web of Science Core Collection (WoSCC) as the sole data source. WoSCC provides standardized metadata and complete cited-reference fields, which are required for co-citation and citation-burst analyses and are fully compatible with CiteSpace and VOSviewer (Xiao et al., 2024b). Using a single database also avoids duplicate records and field-mapping inconsistencies across platforms, which improves reproducibility. We acknowledge that Scopus or Dimensions may increase coverage, but they require additional harmonization steps and can yield non-comparable records depending on document types and indexing policies.

A comprehensive search was conducted in WoSCC, and the full records were downloaded on February 18, 2025. The analysis time window was set to publications from 1999 to 2024. The search was performed in the Topic field (Title, Abstract, Author Keywords, and Keywords Plus) using the following query: TS=((“autism spectrum disorder” OR “ASD” OR autism OR “disorder, autistic spectrum”) AND (“gut microbiota” OR “gut flora” OR microbiome OR “intestinal microbiota”)). Records were limited to English-language research articles and reviews. Conference proceedings, meeting abstracts, letters, editorial materials, and comments were excluded. All records were exported from WoSCC with “Full Record and Cited References” for downstream analyses.

### 2.2 Data analysis

All retrieved records were imported into the analysis workflow without manual screening because this study focuses on bibliometric mapping rather than evidence synthesis. We performed basic data cleaning to improve name consistency for countries and institutions and to reduce obvious duplicates when present. The final dataset was used consistently across all software tools.

### 2.3 Quantitative analysis

In order to intuitively showcase the analysis results, valid data retrieved from the WoSCC database were fed into CiteSpace (version 6.3) and VOSviewer (version 1.6.18). CiteSpace was harnessed to create visual representations of the collaborative networks involving institutions, authors, keywords, and co-cited references. It uncovered citation surges for both references and keywords, and probed into the present situation, focal points, and evolving trends of the research. Through generating cluster plots and temporal distribution plots, emerging trends in the field could be spotted. VOSviewer, on the other hand, enabled visual analysis of the collaborative networks between different countries and academic journals. At the same time, R software (version 4.1) was put to use for visual analysis concerning the countries of publication, thereby further clarifying the patterns and trends related to global research collaborations.

## 3 Result

### 3.1 Annual trends in publications

**FIGURE 1:**
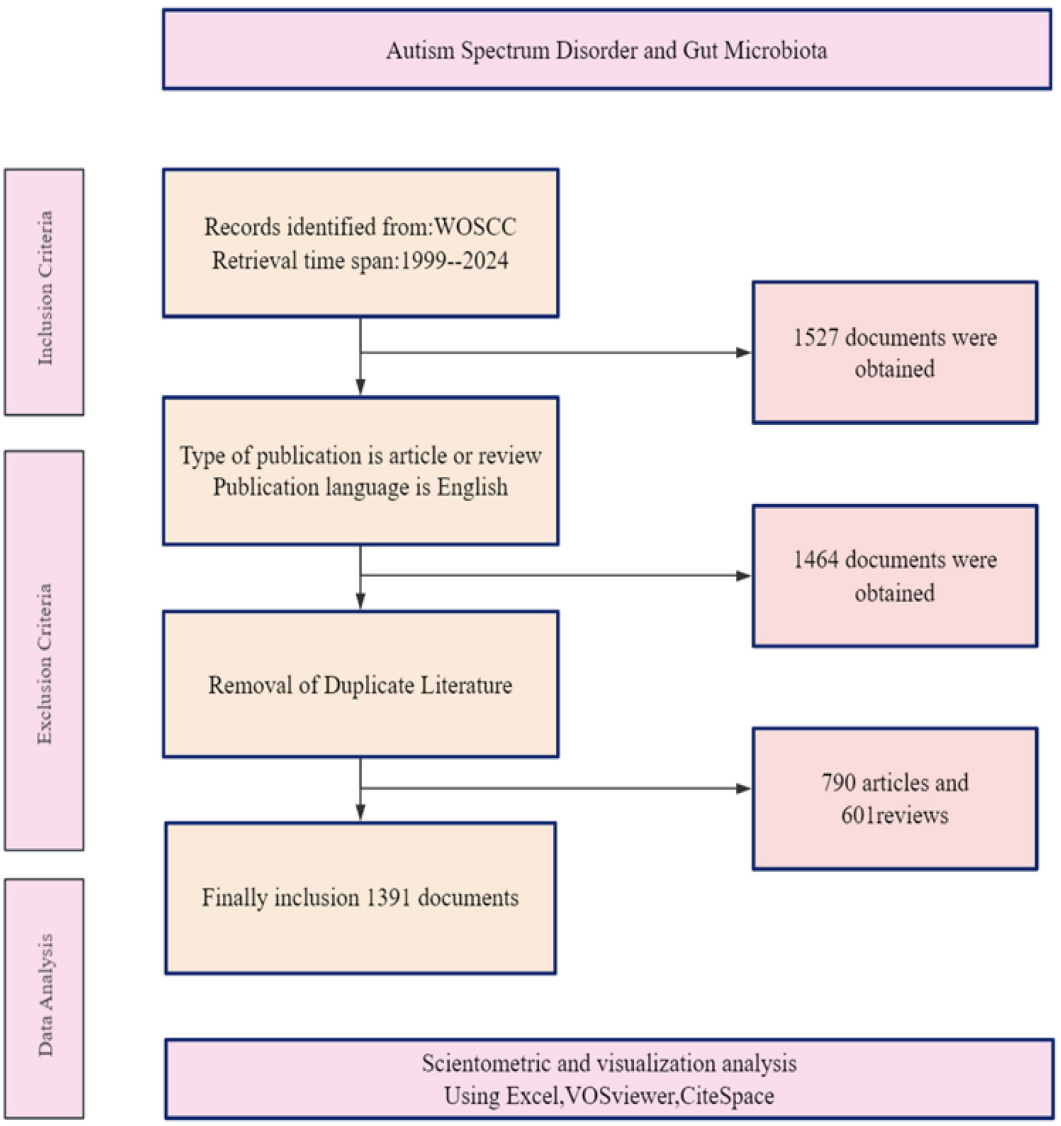
Flow chart for study identification and data preparation. Flow diagram showing the identification, screening, and inclusion of records from the Web of Science Core Collection (WoSCC). Records were retrieved for 1999–2024 and exported on 18 February 2025. Filters were applied to retain English-language research articles and reviews and to remove document types such as conference materials, comments, and letters. The final dataset used for bibliometric analyses comprised 1,391 publications.

**FIGURE 2:**
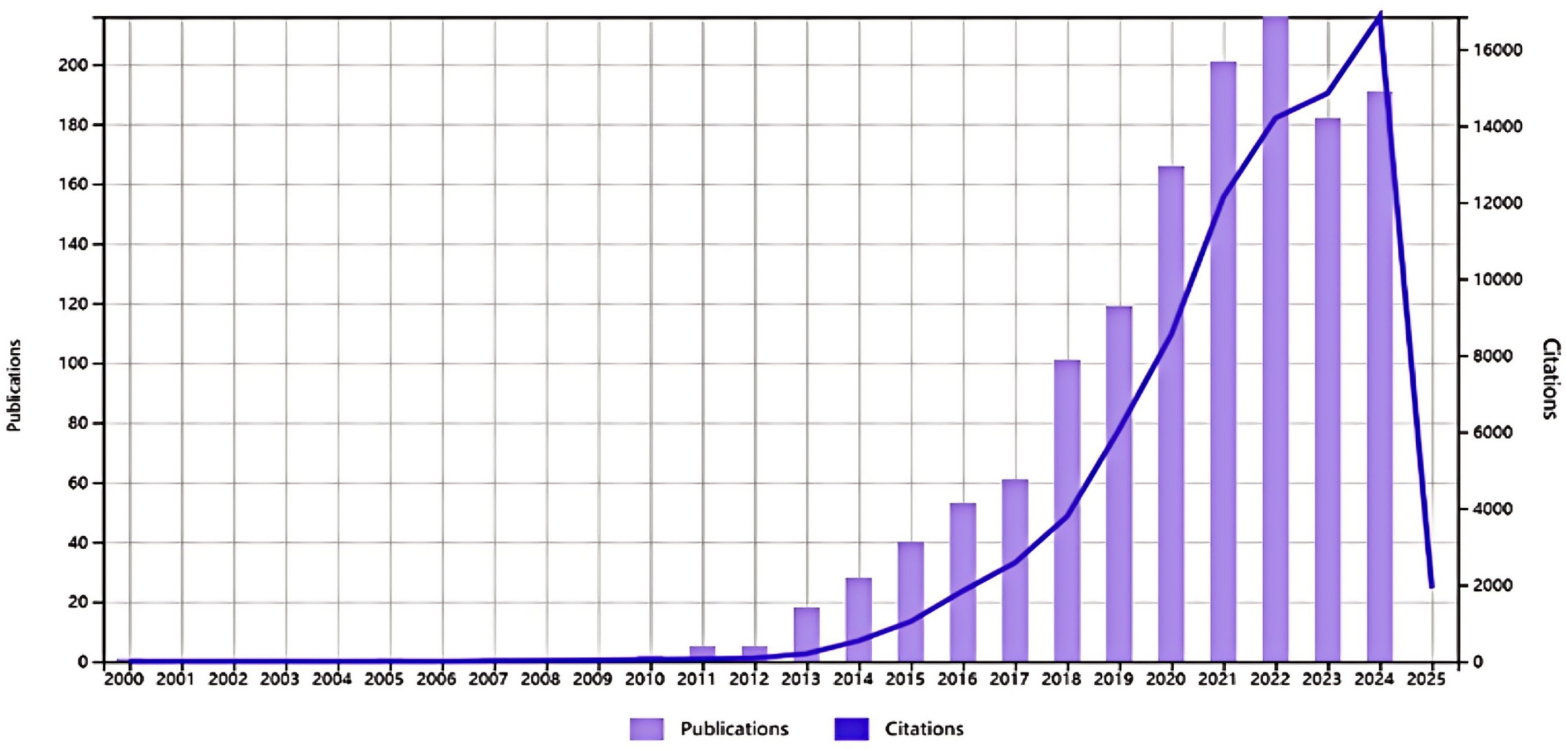
Annual publication output and citation trend. Annual number of WoSCC-indexed publications on ASD and the gut microbiota (bars) and annual citation counts (line) for the included dataset. Values reflect the records retrieved on 18 February 2025; therefore, counts near the endpoint may be influenced by incomplete indexing for the most recent year.

A total of 1,391 studies were included from 1999 to 2024 (Figure 1). As shown in Figure 2, publication output remained low and increased slowly from 1999 to around 2010, followed by a steady rise during the subsequent years. A clear acceleration was observed after 2018, indicating rapid expansion of ASD–gut microbiota research. The annual publication output peaked in 2022 and remained at a high level through 2024 (Figure 2). Citation counts also increased over time and reached their highest level in 2024 (Figure 2), suggesting growing academic attention to this field.

### 3.2 Analysis of publishing countries/regions and institutions

**FIGURE 3:**
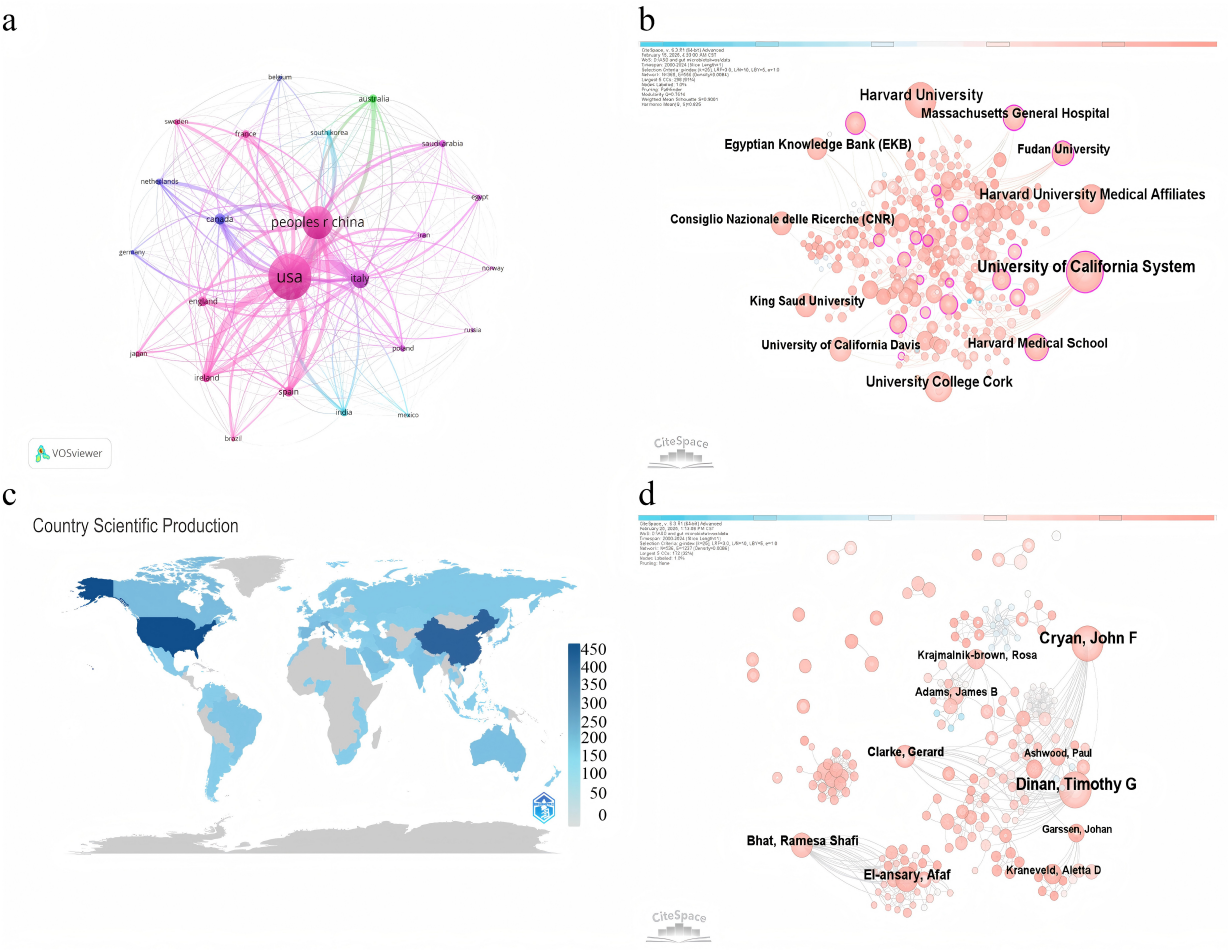
Collaboration landscape across countries, institutions, and authors. **(a)** Country collaboration network generated by VOSviewer. Nodes represent countries/regions. Node size reflects publication output. Links indicate co-authorship relations, and link thickness reflects collaboration strength. Colors indicate community clusters identified by VOSviewer. **(b)** Institution collaboration network generated by CiteSpace. Nodes represent institutions and links indicate inter-institutional collaboration. Node rings are colored by time slice from earlier to more recent years. Purple rims indicate higher betweenness centrality. CiteSpace settings: timespan 2000–2024, slice length 1 year, g-index (*k* = 25), Pathfinder pruning. **(c)** Geographic distribution of publication output by country/region. **(d)** Author collaboration network generated by CiteSpace. Nodes represent authors and links represent co-authorship relations. Visual encodings follow panel **(b)**. CiteSpace settings: timespan 2000–2024, slice length 1 year, g-index (*k* = 25).

The bibliometric analysis included studies from 76 countries and 363 institutions. Figure 3 presents the distribution of publications by country. As shown in Table 1, the United States ranked first with 430 publications (1999–2024). The People’s Republic of China ranked second with 272 publications, followed by Italy with 139 publications. The h-index derived from WoSCC reflects both publication quantity and citation impact. The United States had the highest h-index (86) (Table 1), indicating strong influence in this research area.

**TABLE 1:** Top 10 active countries/regions.

Rank	Country	Publications	Citations	Average citation/publication	H-index
1	USA	430	35551	82.68	86
2	People’s Republic of China	272	8929	30.58	51
3	Italy	139	9924	71.4	46
4	Canada	76	4695	61.78	35
5	Spain	65	2433	37.43	26
6	England	61	3709	60.8	27
7	Ireland	60	12332	205.53	41
8	Australia	49	2891	59	23
9	India	46	988	21.48	15
10	France	44	9190	33.48	20

The country collaboration network is shown in Figure 3a. Each node represents a country, node size is proportional to publication output, and link thickness reflects collaboration strength. The United States and China occupy central positions, with dense collaboration links across the network (Figure 3a).

Table 2 lists the top institutions and their citation performance. In terms of average citations per publication, University College Cork ranked first (52 publications; 232.98 citations per publication). Harvard University had 61 publications (42.05 citations per publication). The University of California System published 75 papers and showed a high average citation rate (151.39 citations per publication). Because the University of California system aggregates multiple campuses, its output may overlap with campus-level records, and the results should be interpreted as an institutional system-level indicator.

**TABLE 2:** Top 10 active institutions.

Rank	Organization	Publications	Citations	Average citation/publication	H-index
1	University of California System	75	11,354	151.39	37
2	Harvard University	61	2,565	42.05	27
3	University College Cork	52	12,115	232.98	40
4	Harvard University Medical Affiliates	46	2,150	46.74	23
5	Harvard Medical School	35	1,522	43.49	17
6	Massachusetts General Hospital	33	1,620	49.09	19
7	University of California Davis	30	1,676	55.87	16
8	Egyptian Knowledge Bank Ekb	28	666	23.79	14
9	King Saud University	27	402	14.89	13
10	Consiglio Nazionale Delle Ricerche Cnr	26	1,205	46.35	17

### 3.3 Co-cited references, authors, and journals

Co-citation analysis reveals influential references and the intellectual structure of the field. Author and journal indicators are summarized here to provide an integrated view of major contributors.

**FIGURE 4:**
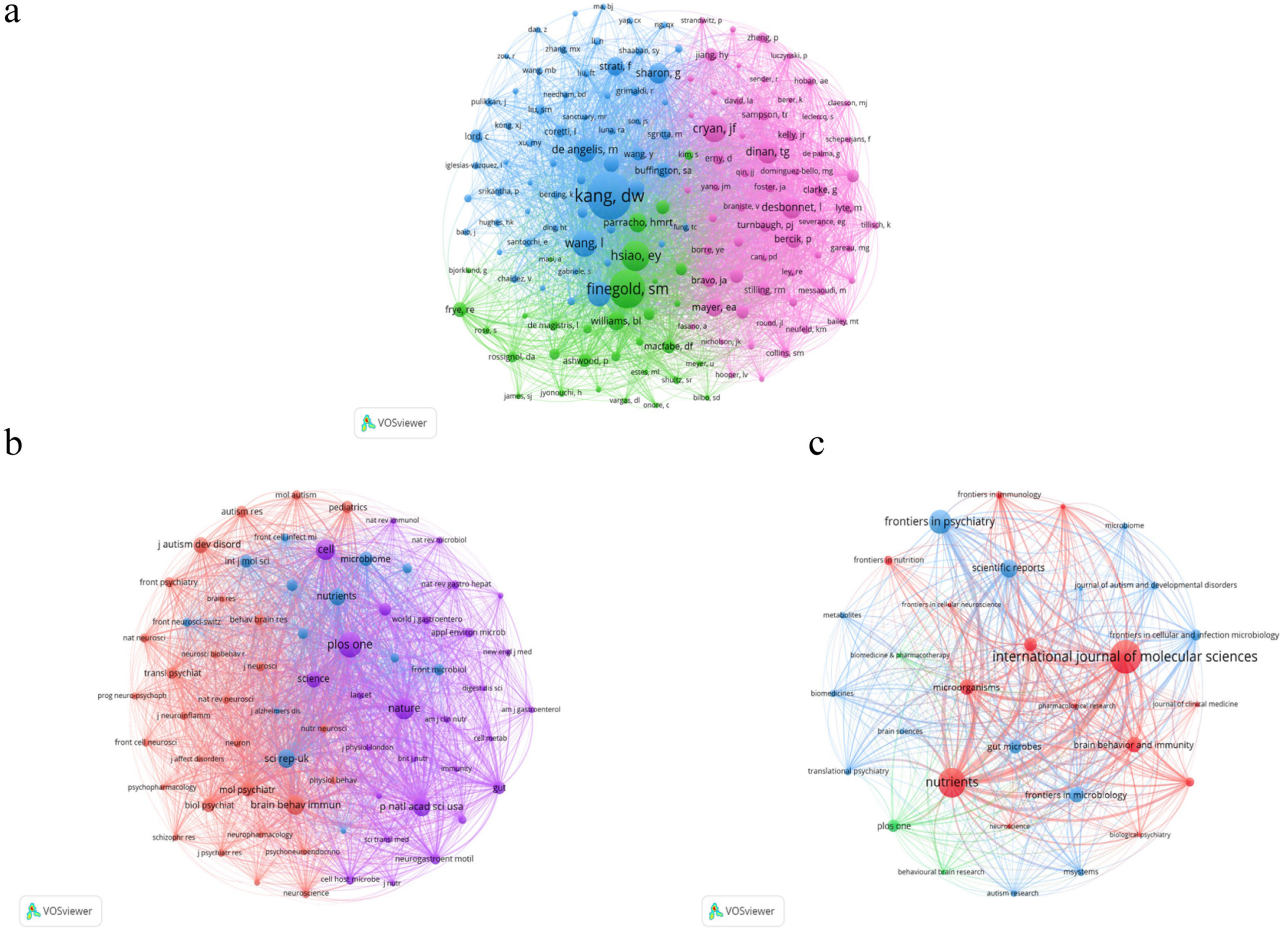
Author and journal networks based on VOSviewer. **(a)** Author network map generated by VOSviewer. Nodes represent authors, node size reflects author influence in the dataset (based on the selected VOSviewer analysis type), and links represent relatedness between authors in the network. Colors indicate clusters. **(b)** Journal network map generated by VOSviewer. Nodes represent journals, node size reflects publication volume in the dataset, and links indicate journal relatedness in the selected VOSviewer analysis. Colors indicate clusters. **(c)** Journal co-citation network generated by VOSviewer. Nodes represent journals and node size reflects co-citation frequency. Links represent co-citation relations, and colors indicate clusters.

Among authors, John F. Cryan (43 publications) and Timothy Dinan (41 publications) were the most productive (Tables 3, 4). Dae-Wook Kang (581 co-citations) and E. Y. Hsiao (507 co-citations) were among the most highly co-cited authors (Table 4), indicating strong influence on the knowledge base of ASD–microbiome research. Betweenness centrality values for authors were generally low, suggesting that the collaboration network is not dominated by a single bridging author but reflects contributions from multiple groups (Figure 4).

**TABLE 3:** Top 10 authors in terms of total publications.

Rank	Author	Publications	Citations	Average citation/publication	H-index
1	Cryan, John F.	43	11,665	271.28	36
2	Dinan, Timothy	41	11,127	271.39	36
3	EL-Ansary, Afaf	21	312	14.86	11
4	Clarke, Gerard	19	6,233	328.05	18
5	Shafi, Ramesa	15	248	16.53	9
6	Kraneveld, Aletta D.	14	626	44.71	9
7	Krajmalnik-Brown, Rosa	13	3,055	235	11
8	MacFabe, Derrick F.	13	1,013	77.92	13
9	Adams, James B.	12	2,480	206.67	10
10	Frye, Richard	11	806	73.27	11

**TABLE 4:** Top 10 co-cited authors in terms of total citations.

Rank	Cited authors	Count	Centrality
1	KANG DW	581	0
2	HSIAO EY	507	0
3	FINEGOLD SM	468	0
4	ADAMS JB	393	0
5	CRYAN JF	393	0
6	DE ANGELISM	359	0
7	WANG L	352	0
9	SHARON G	316	0
10	KANG DW	298	0
11	STRATI F	298	0

At the journal level, International Journal of Molecular Sciences (52 articles) and Nutrients (46 articles) had the highest publication output (Table 5). In co-citation analysis, PLOS ONE (1,092 co-citations), Cell (945), Nature (879), and Scientific Reports (855) were the most frequently co-cited journals (Table 6), indicating that foundational evidence is widely distributed across interdisciplinary outlets.

**TABLE 5:** Top 10 journals in terms of total publications.

Rank	Journals rank	Publications	Citations	Average citation/publication	H-index	Journal IF (2023)
1	International Journal of Molecular Sciences (Q1)	52	1,453	27.94	21	4.9
2	Nutrients (Q2)	46	2,620	56.96	22	3.8
3	Frontiers in Psychiatry (Q2)	37	969	26.19	15	4.2
4	Scientific Reports (Q1)	28	1,417	50.61	15	4.0
5	Frontiers in Microbiology (Q2)	26	2,714	104.38	14	4.0
6	Brain Behavior and Immunity (Q1)	24	1,894	78.92	16	8.8
7	Microorganisms (Q2)	24	2,333	97.21	13	4.1
8	Frontiers in Neuroscience (Q2)	23	779	33.87	12	3.2
9	Gut Microbes (Q1)	22	755	34.32	11	12.2
10	Plos One (Q1)	19	2,497	131.42	16	2.9

**TABLE 6:** Top 10 co-cited journals in terms of total citations.

Rank	Cited references	Count	Centrality
1	PLOS ONE	1092	0
2	CELL	945	0
3	NATURE	879	0
4	SCI REP-UK	855	0
5	P NATL ACAD SCI USA	840	0
6	BRAIN BEHAV IMMUN	794	0
7	MICROBIOME	728	0
8	NUTRIENTS	697	0
9	SCIENCE	686	0
10	BIOL PSYCHIAT	686	0

**FIGURE 5:**
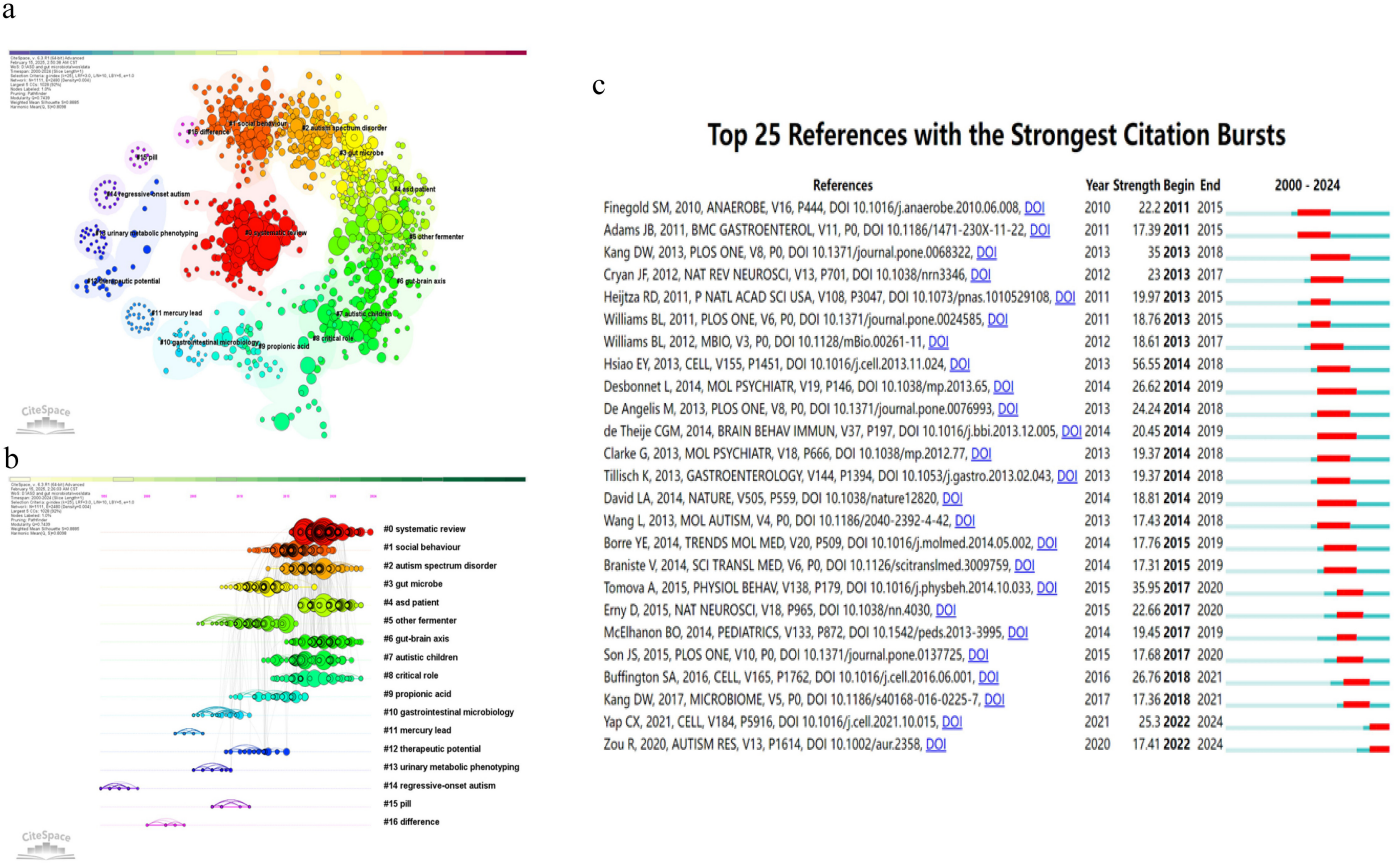
Reference co-citation structure and citation burst detection (CiteSpace). **(a)** Co-cited reference network generated by CiteSpace. Nodes represent cited references and node size reflects co-citation frequency. Links represent co-citation relations. Node rings are colored by time slice from earlier to more recent years. Purple rims indicate higher betweenness centrality. **(b)** Co-citation clusters labeled using the log-likelihood ratio (LLR) algorithm. Cluster labels summarize the main topics represented by each cluster. **(c)** Top 25 references with the strongest citation bursts. Red segments indicate burst periods, and blue segments indicate non-burst periods across the study timeline. CiteSpace settings for panels **(a–c)**: timespan 2000–2024, slice length 1 year, g-index (*k* = 25), Pathfinder pruning.

For co-cited references, Sharon et al. (2019) ranked first with 256 co-citations (Table 7). The co-citation clustering identified 17 reference clusters (Figures 5a,b), including #0 systematic review, #1 social behavior, #2 autism spectrum disorder, #3 gut microbe, #6 gut-brain axis, #9 propionic acid, and #12 therapeutic potential, reflecting the thematic structure from descriptive clinical observations to mechanism- and intervention-related studies. In addition, the top citation bursts (Figure 5c) highlight recent influential references, including Yap et al. (2021) and Zou et al. (2020), both showing strong bursts from 2022 to 2024.

**TABLE 7:** Top 10 co-cited documents.

Rank	Title	First author	Cited Journals	Cited journals	Count	Centrality
1	Human Gut Microbiota from Autism Spectrum Disorder Promote Behavioral Symptoms in Mice	Gil Sharon	Sharon G, 2019, CELL, V177, P1600, DOI 10.1016/j.cell.2019.05.004	Cell	256	0
2	Microbiota Transfer Therapy alters gut ecosystem and improves gastrointestinal and autism symptoms: an open-label study	Dae-Wook Kang	Kang DW, 2017, MICROBIOME, V5, P0, DOI 10.1186/s40168-016-0225-7	Microbiome	237	0
3	Long-term benefit of Microbiota Transfer Therapy on autism symptoms and gut microbiota	Dae-Wook Kang	Kang DW, 2019, SCI REP-UK, V9, P0, DOI 10.1038/s41598-019-42183-0	Sci Rep	204	0
4	New evidences on the altered gut microbiota in autism spectrum disorders	Francesco Strati	Strati F, 2017, MICROBIOME, V5, P0, DOI 10.1186/s40168-017-0242-1	Microbiome	198	0
5	Mechanisms Underlying Microbial-Mediated Changes in Social Behavior in Mouse Models of Autism Spectrum Disorder	Martina Sgritta	Sgritta M, 2019, NEURON, V101, P246, DOI 10.1016/j.neuron.2018.11.018	Neuron	162	0
6	Altered gut microbiota and short chain fatty acids in Chinese children with autism spectrum disorder	Simeng Liu	Liu SM, 2019, SCI REP-UK, V9, P0, DOI 10.1038/s41598-018-36430-z	Scientific Reports	150	0
7	Emerging Roles for the Gut Microbiome in Autism	Helen E. Vuong	Vuong HE, 2017, BIOL PSYCHIAT, V81, P411, DOI 10.1016/j.biopsych.2016.08.024	Biological Psychiatry	143	0
8	Spectrum DisorderThe microbiome in ASD”	John F. Cryan	Cryan JF, 2019, PHYSIOL REV, V99, P1877, DOI 10.1152/physrev.00018.2018	Physiological Reviews	141	0
9	The Microbiota-Gut-Brain Axis	Fattorusso A	Fattorusso A, 2019, NUTRIENTS, V11, P0, DOI 10.3390/nu11030521	Nutrients	140	0
10	Autism, Gastrointestinal Symptoms and Modulation of Gut Microbiota by Nutritional Interventions	Buffington SA	Buffington SA, 2016, CELL, V165, P1762, DOI 10.1016/j.cell.2016.06.001	Cell	133	0

### 3.4 Keyword co-occurrence and keyword prominence

Table 8 lists the top active keywords extracted from the 1,391 publications. The most frequent keywords included “gut microbiota,” “autism spectrum disorder,” “children,” and “brain,” indicating that ASD core phenotype, developmental stage, and gut microbial ecology are central foci of the field. Keyword co-occurrence analysis also reflects conceptual connectivity. Some terms with lower frequency still showed relatively higher centrality, suggesting their role as connectors between themes in the network.

**TABLE 8:** Top 20 active keywords.

Rank	Keywords	Count	Centrality
1	Gut microbiota	591	0.02
2	Autism spectrum disorder	416	0.02
3	Children	367	0.12
4	Brain	187	0.05
5	Intestinal microbiota	169	0.01
6	Autism spectrum disorders	157	0.03
7	Gut-brain axis	152	0.03
8	Chain fatty acids	151	0.03
9	Autism	134	0.03
10	Gut microbiome	120	0.04
11	Health	118	0.07
12	Behavior	111	0.03
13	Gastrointestinal symptoms	101	0.03
14	Parkinson’s disease	100	0.05
15	Oxidative stress	87	0.04
16	Fecal microbiota	82	0.02
17	Spectrum disorder	81	0.02
18	Microbiota	81	0.03
19	Irritable bowel syndrome	78	0.04
20	Spectrum disorders	77	0.1

**FIGURE 6:**
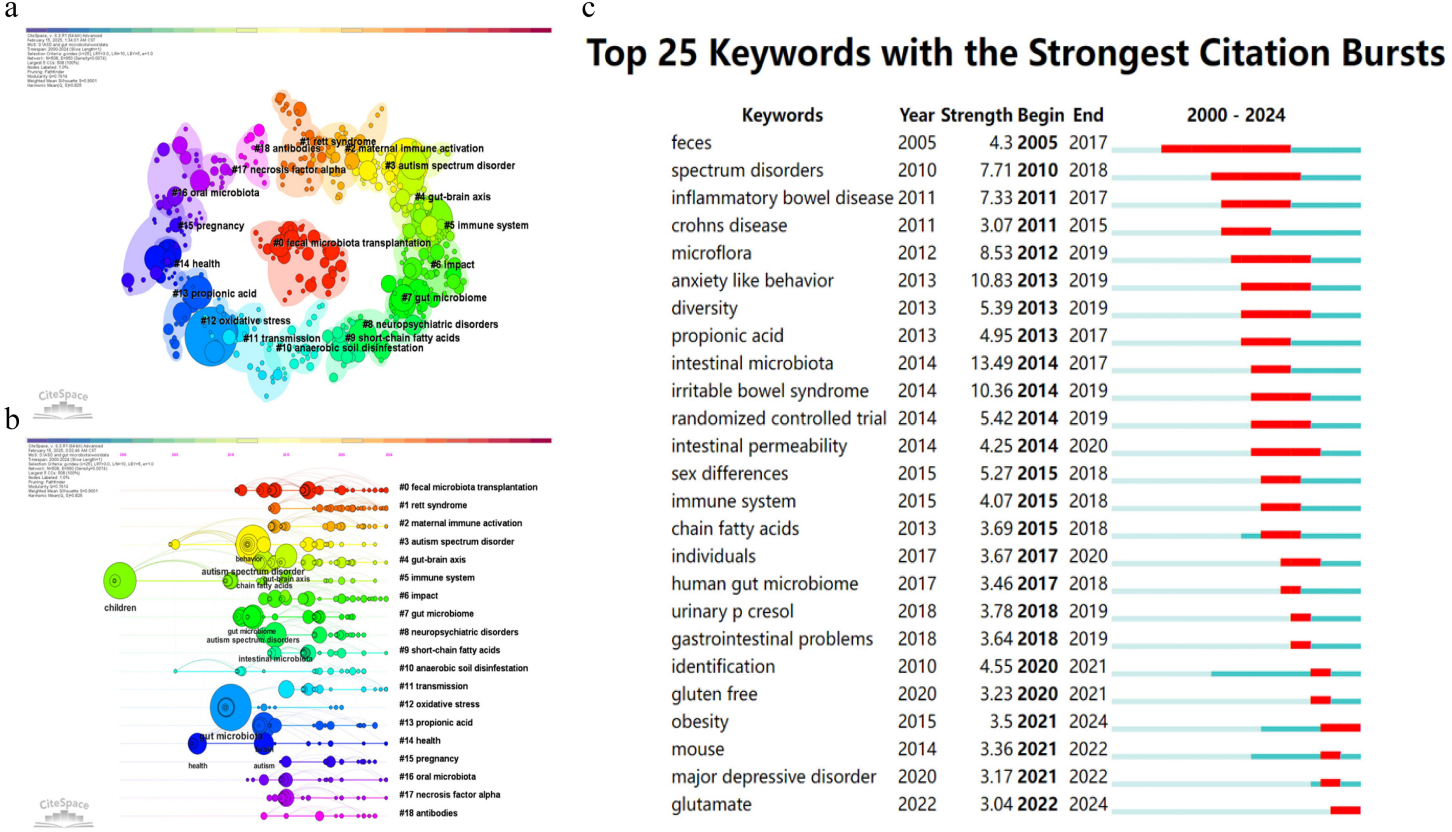
Keyword co-occurrence structure, timelines, and burst keywords (CiteSpace). **(a)** Keyword co-occurrence network generated by CiteSpace. Nodes represent keywords and node size reflects occurrence frequency. Links represent co-occurrence relations. Node rings are colored by time slice from earlier to more recent years. Purple rims indicate higher betweenness centrality. **(b)** Keyword clustering timeline view. Each horizontal line represents a keyword cluster. The position of nodes indicates the time periods when keywords were active within each cluster. Cluster labels summarize the conceptual focus of each cluster, including fecal microbiota transplantation, Rett syndrome, and maternal immune activation. **(c)** Top 25 keywords with the strongest citation bursts. Red segments indicate burst periods across the timeline. CiteSpace settings for panels **(a–c)**: timespan 2000–2024, slice length 1 year, g-index (*k* = 25), Pathfinder pruning.

**FIGURE 7:**
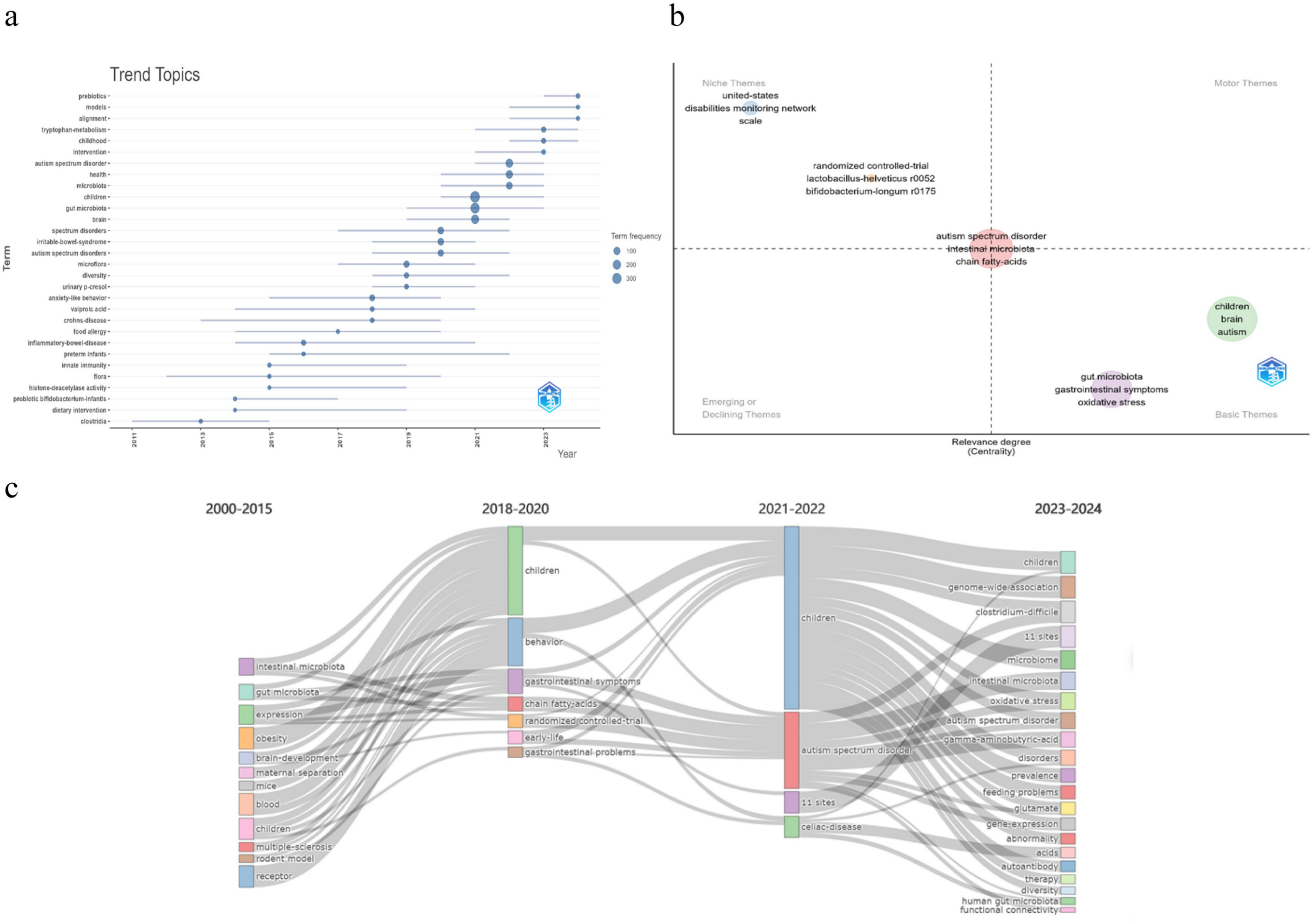
Keyword-based topic trends and thematic evolution. **(a)** Trend topics over time based on keyword dynamics. **(b)** Thematic map showing themes positioned by centrality (relevance) and density (development). Themes in the upper-right quadrant represent motor themes, and themes in the lower-right quadrant represent basic themes. **(c)** Thematic evolution diagram across time slices (2000–2015, 2018–2020, 2021–2022, 2023–2024), showing how major themes split, merge, or persist over time.

The keyword clustering analysis identified 19 clusters (from #0 to #18) (Figures 6, 7). The three most prominent clusters were #0 fecal microbiota transplantation, #1 Rett syndrome, and #2 maternal immune activation (Figure 6b), representing intervention-focused research, syndromic neurodevelopmental conditions, and developmental immune-mechanism themes. Burst keyword analysis (Figure 6c) showed several emerging topics in recent years. “Obesity” showed a burst from 2021 to 2024; “major depressive disorder” showed a burst from 2021 to 2022; and “glutamate” showed a burst from 2022 to 2024, indicating expanding interest in metabolic, psychiatric, and neurotransmitter-related dimensions of ASD–microbiome research.

## 4 Discussion

### 4.1 General information

This study mapped 1,391 WoSCC-indexed publications on ASD and the gut microbiota from 1999 to 2024. The annual output increased slowly before 2010 and rose sharply after 2018, indicating sustained growth of this interdisciplinary field (Figure 2). Country and institution networks showed that the United States and China were the main contributors and collaboration hubs (Figure 3; Table 1). University College Cork, Harvard University, and the University of California System were among the most productive or most cited institutions (Table 2).

Author and journal analyses further described the knowledge landscape. Cryan and Dinan were the most prolific authors, while Kang and Hsiao were among the most co-cited, reflecting their strong influence on the field’s intellectual structure (Tables 3, 4). Journals with high output included International Journal of Molecular Sciences and Nutrients, while highly co-cited journals included PLOS ONE, Cell, and Nature (Tables 5, 6). These patterns suggest that ASD–microbiome research is published across both specialized microbiome outlets and broad interdisciplinary journals.

Co-citation and keyword analyses clarified how the field has evolved. The top co-cited documents (Table 7) show that the intellectual core is anchored by experimental evidence for microbiome–behavior links and by translational work on microbiome-targeted interventions. Landmark studies include Sharon et al. (2019) and microbiota transfer therapy studies by Kang et al. (2017, 2019). Several other papers were also highly co-cited (Buffington et al., 2016; Strati et al., 2017; Liu et al., 2019; Sgritta et al., 2019; Cryan et al., 2019; Fattorusso et al., 2019), reflecting increased attention to mechanisms and intervention-oriented research.

The keyword timeline clustering in Figure 6 further supports this evolution. Three prominent clusters were #0 fecal microbiota transplantation, #1 Rett syndrome, and #2 maternal immune activation. These clusters span intervention research, syndromic neurodevelopmental conditions, and prenatal or early-life immune mechanisms. Table 8 also shows that “gut-brain axis” and “short chain fatty acids” are central recurring concepts, alongside “gastrointestinal symptoms” and “oxidative stress,” indicating sustained interest in gut physiology and systemic biology. Keywords such as “obesity,” “major depressive disorder,” and “glutamate” emerged recently and may indicate increasing convergence with metabolic and broader psychiatric domains.

### 4.2 Biological interpretation of major themes in the literature

The clusters and timelines in Figure 6, together with the top co-cited papers (Table 7) and burst keywords (Table 8), outline how ASD–microbiome research has shifted in both questions and methods. Early work emphasized cross-sectional differences and gastrointestinal comorbidity. Later studies increasingly tested mechanisms and intervention relevance, which is reflected by the rising prominence of the gut–brain axis, microbial metabolites, and treatment-related themes.

Across clusters, the co-cited core literature supports a shift from descriptive comparisons to mechanism-oriented hypotheses. Sharon et al. (2019) is a key experimental anchor, showing that ASD-associated microbiota can influence behavioral phenotypes in germ-free mice (Sharon et al., 2019). Mechanistic frameworks that shaped later work describe how microbial communities can affect neurodevelopment and social behavior through defined pathways in animal models (Sgritta et al., 2019). Synthesis work by Cryan et al. (2019) and Fattorusso et al. (2019) helped consolidate the gut-brain axis as an organizing framework that integrates microbial ecology, immune signaling, metabolism, and neural function.

Metabolite- and immune-related mechanisms form a second theme that links multiple clusters. Table 8 shows “short chain fatty acids” as a recurring concept, consistent with mechanistic discussions that connect SCFAs to neuroimmune function and synaptic physiology (Fung et al., 2017; Dalile et al., 2019). Related work also highlights tryptophan catabolites and metabolite-linked inflammatory pathways as candidate mediators (Morris et al., 2017). Dietary modulation sits within this metabolite-centered theme as a practical lever. Buffington et al. (2016), which appears among the highly co-cited papers in Table 7, connects diet-related modulation to behavioral outcomes in experimental settings. Liu et al. (2019) provides a cohort-level anchor that links microbiome profiles and metabolite-related readouts in ASD. Together, these works support a biologically plausible pathway from diet to microbial metabolism to host signaling, while also emphasizing that it requires better control of dietary confounding and more consistent metabolite measurement.

Intervention-oriented research is represented by cluster #0 fecal microbiota transplantation in Figure 6 and by the prominence of microbiota transfer therapy studies in Table 7. Kang et al. (2017, 2019) are central anchors for this cluster and reflect the field’s move toward testing whether modifying microbial communities can improve gastrointestinal symptoms and ASD-relevant outcomes. This cluster also connects to recurring keywords related to gastrointestinal symptoms and health in Table 8. At the same time, heterogeneity remains a limiting factor. Intervention studies differ in design, participant phenotyping, and outcome definitions. Larger trials with clearer responder definitions, stronger controls, and clinically meaningful endpoints are needed to assess efficacy, safety, and durability across ASD subgroups.

A developmental timing theme is supported by cluster #2 maternal immune activation in Figure 6 and by the centrality of Hsiao in the knowledge structure (Table 4). Hsiao et al. (2013) provides a widely cited experimental basis for microbiome-dependent immune pathways that can shape neurobehavioral phenotypes during development (Hsiao et al., 2013). Early life colonization, pregnancy-related factors, and transmission-related concepts can be viewed as extensions of this life course perspective, where microbial assembly and immune exposures act as upstream determinants rather than downstream correlates. This framing helps explain why child and developmental terms remain prominent in the keyword landscape. Progress in this area requires longitudinal mother-infant cohorts, standardized sampling, and exposure measurement, including antibiotic use, feeding, and infections, together with harmonized analytic pipelines.

Finally, the recent appearance of “obesity,” “major depressive disorder,” and “glutamate” in Table 8 suggests expanding links to metabolic phenotypes, psychiatric dimensions, and neurotransmitter-related hypotheses. These terms likely reflect a convergence of ASD microbiome research with broader work on metabolism, inflammation, and neural signaling, and they also mirror clinical realities of comorbidity. Their presence should be interpreted as a signal of shifting research agendas rather than as evidence of established causal pathways. Studies will need clearer phenotyping, confounder control, and designs that can separate ASD-specific effects from shared comorbidity mechanisms (Długosz et al., 2025).

Overall, the clusters and timelines in Figure 6, the co-cited anchors in Table 7, and the keyword dynamics in Table 8 collectively depict a field that increasingly adopts systems-level explanations and translational questions. These bibliometric signals indicate when themes gained prominence, while causal and mechanistic claims depend on the underlying primary evidence.

### 4.3 Implications, gaps, and future directions

The bibliometric mapping highlights several gaps that likely limit progress. Cross-study comparability remains a major issue. Sampling protocols, sequencing platforms, bioinformatic pipelines, and statistical models vary widely. This variation reduces reproducibility and complicates meta-level synthesis. Longitudinal designs remain less common than cross-sectional studies, which limits inference about temporal ordering. Multi-omics integration is increasing, but it is still not routine in many cohorts.

Future work should prioritize three directions. First, longitudinal cohorts with standardized protocols are needed to clarify developmental dynamics and reduce methodological heterogeneity. Second, integrative multi-omics studies should link microbiome profiles with immune, metabolic, and neural measures using transparent and reproducible analytic workflows. Third, microbiome-targeted interventions should be tested in rigorous clinical trials with clear responder definitions and clinically meaningful endpoints.

### 4.4 Strengths and limitations

This study provides a structured bibliometric mapping of ASD–gut microbiota research published between 1999 and 2024, based on 1,391 WoSCC-indexed records. Using WoSCC enabled access to standardized metadata and complete cited-reference fields, which supports co-citation and citation-burst analyses and improves reproducibility.

We combined complementary tools (CiteSpace, VOSviewer, and bibliometrix in R) to characterize productivity, collaboration networks, knowledge structure, and evolving keywords in a consistent workflow. By integrating co-citation anchors, timeline clustering, and burst keywords, the study offers an evidence-based overview of how major themes emerged and consolidated over time.

Several limitations should be considered when interpreting rankings, clusters, and temporal patterns. First, WoSCC was used as the sole data source and records were limited to English-language research articles and reviews, which may reduce coverage compared with multi-database searches. Second, records were imported without manual screening because the goal was bibliometric mapping rather than evidence synthesis; therefore, some topic noise may remain despite basic cleaning. Third, bibliometric indicators reflect research attention and citation behavior. They do not evaluate study quality and they can be influenced by citation lag, document type, and field-specific practices. Fourth, clustering and network outputs may vary with software parameter settings and author or institution name disambiguation. These limitations do not alter the descriptive value of the mapping, but they indicate that biological and clinical conclusions should be grounded in primary studies rather than inferred from bibliometric signals alone.

## 5 Conclusion

Research on the gut microbiota in ASD has expanded rapidly since 2018, reflecting sustained growth of an interdisciplinary field. Our bibliometric mapping of 1,391 WoSCC-indexed publications (1999–2024) shows increasing global output, active international collaboration led by the United States and China, and a knowledge structure anchored by highly co-cited experimental and translational work. The thematic landscape, reflected by keyword clusters and timelines, highlights intervention-focused research (fecal microbiota transplantation), syndromic neurodevelopmental conditions (Rett syndrome), and developmental immune mechanisms (maternal immune activation). Recurrent themes include the gut–brain axis, microbial metabolites such as short-chain fatty acids, gastrointestinal symptoms, and oxidative stress.

At the same time, ASD heterogeneity and methodological variation across cohorts and analytic pipelines continue to limit cross-study comparability and causal inference. Emerging burst terms, including obesity, major depressive disorder, and glutamate, indicate that ASD–microbiome research is increasingly intersecting with metabolic phenotypes, psychiatric dimensions, and neurotransmitter-related hypotheses. These signals point to an expanding research agenda, but they do not resolve mechanism or clinical utility on their own.

Future studies should prioritize standardized sampling and analysis workflows, well-designed longitudinal cohorts with careful exposure measurement, and integrative multi-omics approaches that link microbiome profiles to immune, metabolic, and neural readouts. Microbiome-targeted interventions, including dietary modulation, probiotics or synbiotics, and fecal microbiota transplantation, should be tested in rigorous trials with clear phenotyping, responder definitions, and clinically meaningful endpoints to support translation toward more actionable strategies for ASD.

## Data Availability

The original contributions presented in this study are included in this article/supplementary material, further inquiries can be directed to the corresponding authors.
